# The Role of miRNAs in Zearalenone-Promotion of TM3 Cell Proliferation

**DOI:** 10.3390/ijerph16091517

**Published:** 2019-04-29

**Authors:** Wanglong Zheng, Wentong Fan, Nannan Feng, Nanyan Lu, Hui Zou, Jianhong Gu, Yan Yuan, Xuezhong Liu, Jianfa Bai, Jianchun Bian, Zongping Liu

**Affiliations:** 1 College of Veterinary Medicine, Yangzhou University, Yangzhou 225009, Jiangsu, China; zhengwanglong@163.com (W.Z.); f1256963688@163.com (W.F.); fengnannan8@outlook.com (N.F.); zouhui@yzu.edu.cn (H.Z.); jhgu@yzu.edu.cn (J.G.); yuanyan@yzu.edu.cn (Y.Y.); Liuxuezhong68@163.com (X.L.); 2 Jiangsu Co-innovation Center for Prevention and Control of Important Animal Infectious Diseases and Zoonoses, Yangzhou 225009, Jiangsu, China; 3 Joint International Research Laboratory of Agriculture and Agri-Product Safety of the Ministry of Education of China, Yangzhou University, Yangzhou 225009, Jiangsu, China; 4 Kansas State Veterinary Diagnostic Laboratory, Kansas State University, 1800 Denison Avenue, Manhattan, KS 66506, USA; nanyan@vet.k-state.edu (N.L.); jbai@vet.k-state.edu (J.B.)

**Keywords:** zearalenone (ZEA), cell proliferation, MicroRNA, cell cycle, TM3 cell

## Abstract

Zearalenone (ZEA) is a non-steroidal estrogen mycotoxin produced by several *Gibberella* and *Fusarium* species. Accumulating evidence has indicated that ZEA strongly stimulates cell proliferation. However the detailed molecular and cellular mechanisms of ZEA-mediated induction of cell proliferation have not yet been completely explained. The aim of this study was to detect the role of miRNAs in ZEA-mediated induction of cell proliferation. The effects of ZEA on cell proliferation were assessed using a cell counting kit assay and xCELLigence system. Micro-RNA sequencing was performed after treatment of TM3 cells with ZEA (0.01 μmol/L) for different time periods (0, 2, 6 and 18 h). Cell function and pathway analysis of the miRNA target genes were performed by Ingenuity Pathway Analysis (IPA). We found that ZEA promotes TM3 cell proliferation at low concentrations. miRNA sequenceing revealed 66 differentially expressed miRNAs in ZEA-treated cells in comparison to the untreated control (*p* < 0.05). The miRNA sequencing indicated that compared to control group, there were 66 miRNAs significant change (*p* < 0.05) in ZEA-treated groups. IPA analysis showed that the predicated miRNAs target gene involved in cell Bio-functions including cell cycle, growth and proliferation, and in signaling pathways including MAPK and RAS-RAF-MEK-ERK pathways. Results from flow cytometry and Western Blot analysis validated the predictions that ZEA can affect cell cycle, and the MAPK signaling pathway. Taking these together, the cell proliferation induced ZEA is regulated by miRNAs. The results shed light on the molecular and cellular mechanisms for the mediation of ZEA to induce proliferation.

## 1. Introduction

Zearalenone (ZEA) mainly comes from foods and feeds contaminated by several species of *Gibberella* and *Fusarium* [1]. These fungi contaminate cereal grains, including maize, wheat, sorghum, barley, and oats, and produce ZEA in the farm and field and, or during the period of harvesting and storage at a low temperature and high humidity [2,3,4]. In recent years, several studies have suggested that the structure of ZEA can be modified by microorganisms, plants, animals and humans via glycosylation, sulfation, and acetylation of trichothecenes [5]. The metabolism of ZEA can be divided into two phases including phase-I metabolism and phase-II metabolism. At the phase-I, ZEA was catalyzed by 3α-hydroxysteroid dehydrogenase (3α-HSD) or 3β-hydroxysteroid dehydrogenase (3β -HSD) and transformed into α-zearalenol (α-ZEA), β-zearalenol (β-ZEA), zearalanone (ZAN), α-zearalanol (α-ZAL) and β-zearalanol (β-ZAL) and all of which were subsequently conjugated to glucuronic acid [6]. At the phase-II these metabolites were glucuronidated and sulfated [1]. Accumulating data has revealed that ZEA could impair reproductive ability and perturb the production and development of sperms and oocytes in humans and animals [7,8,9]. Recently several studies have shown that ZEA not only cause cell death but also stimulates cell proliferation at different cells [1]. ZEA, at low concentrations, strongly stimulates the proliferation of MCF-7 cells in at low concentration [10]. ZEA could stimulate T47D cells growth and compared to the control group, the rate of growth was 2-fold in the 10^−8^ M group [11]. ZEA, at a low concentration, enhanced cell proliferation of a colon carcinoma cell line [12]. It has also been suggested that α-ZAL can increase the proliferation of bone marrow stromal cells and of granulosa cells [13,14].

MicroRNAs are a sort of small single-stranded RNAs that can modulate the expression genes through binding to their targeted mRNAs [15]. Many studies have indicated that aberrant expression of miRNAs is involved in the processes of cell proliferation and invasion [16,17]. Mirco-RNAs exert crucial roles in numerous processes including cell apoptosis and proliferation [18]. Studies have shown that miRNAs could promote the transition through the G1/S phase by inhibiting the expression of Retinoblastoma protein, and also could directly regulate the expression of regulatory molecules, such as cyclin E-CDK2 and p21/Cip1 in mouse embryonic stem cells of mouse [19].

As is well known, Leydig cells exert significant roles in regulating the synthesis of sperms and testosterones [20,21]. The agent that could disturb the viability or function of Leydig cells might simultaneously alter the testicular functions [22]. However, the molecular and cellular mechanism of ZEA can promote cell proliferation in Leydig cells is currently unclear. In the current study, high-throughput RNA sequencing was performed to detect the effects of ZEA on miRNAs in Leydig cells. Cellular functions and pathways regulating the differentially expressed genes were analyzed and predicted to elucidate the molecular and cellular mechanism of ZEA-mediated induction of cell proliferation.

## 2. Material and Methods

### 2.1. Reagents and Antibodies

Mouse TM3 cells (Leydig cells) were obtained from the Chinese Academy of Sciences (Shanghai, China); Zearalenone was obtained from Sigma Aldrich (St. Louis, MO, USA); DMEM-F12 medium, horse serum (HRS) and fetal bovine serum (FBS) were purchased from Gibco (Grand Island, NY, USA); the cell cycle assay and Annexin V/propidium iodide assay were obtained from Becton Dickinson Company (Franklin Lakes, NJ, USA); the Enhanced BCA Protein Assay Kit (P0010) was purchased from Beyotime (Shanghai, China). Polyclonal antibodies against α-Actin (8547) Cyclin-D1 (2978), ERK1/2 (4695S), P-ERK1/2 (54240), JNK1/2 (9252S), P-JNK1/2 (4668S), P-P38 (4511) P38 (8690) were acquired from Cell Signaling Technology (Boston, MA, USA); the antibody against CDK4 (ab199728) was obtained from Abcam (Cambridge, MA, USA).

### 2.2. Cell Culture

TM3 cells were cultivated in DMEM/F-12 medium supplemented with 5% HRS, 2.5% FBS, 150 mg/L of L-glutamine, 1.5 g/L of NaHCO_3_ and 100 IU/mL of penicillin at 37 °C with 5% CO_2_. 

### 2.3. Cell Proliferation Analysis 

Cell viability was analyzed using cell counting kit-8 assay (CCK-8) assay kit. TM3 cells were plated in the 96 well culture plates. TM3 cells were exposed to ZEA (0, 0.01, 0.02, 0.03, 0.05, 0.1 and 0.5 μmol/L) for 24 h. 2 h before the end of treatment, CCK-8 solution was added to the cells. The micro-plate absorbance reader was used to detect the optical density (OD) of each well at 450 nm. Cell proliferation was monitored by using the xCELLigence real-time cell analysis (RTCA). The TM3 cell proliferation index was assessed every 15 min. TM3 cells were treated with ZEA at concentrations of ZEA 0, 0.01, 0.02, 0.03, 0.05, 0.1, 0.5 μmol/L. 

### 2.4. Analyzing the Differential Expression MicroRNA

Total RNA was extracted by using the QIAzol Lysis Reagent (QIAGEN, Dusseldorf, Germany) after the cells were treated, and the aqueous phase was removed through a Phase-Lock Gel column after centrifugation. Total RNA was purified by RNeasy Micro Kit and the concentration of RNA was detected by using a NanoDrop^®^ ND2000 spectrophotometer (Thermo Fisher Scientific, Waltham, MA, USA). The differential microRNA expressions were analyzed by the Illumina Hi-Seq 2000 Platform at the Beijing Genomics Institute (BGI, Shengzhen, China). Polyadenylated RNA was purified from the total RNA samples by using magnetic beads conjugated with Oligo dT and according to the Illumina TruSeq RNA Sample Preparation Kit v3 prepared single-end sequencing. 

### 2.5. Analyzing Cell Cycle Distribution 

After TM3 cells were treated with 0.01 μmol/L ZEA for 2, 6, 9 and 18 h, cells were collected, and 70% ethanol was added slowly to fix the cells. Fixed cells were washed with PBS twice and then stained for 30 min at 25 °C. After filtered, the cell cycle distribution was analyzed by the flow cytometry.

### 2.6. Confirmation of miRNA Expression by Quantitative RT-PCR

Differential expressions of miRNA including miR-21a-5p, miR-10a-5p, miR-10b-5p, miR-7a-5p, let-7c-5p were validated by using RT-qPCR. Prime Script RT reagent kit (TAKARA, Kyoto, Japan) was used to respectively elongate miRNAs. Reverse transcription was performed for 60 min at 37 °C. PCR analysis was done in triplicates. The gene U6 was used as an internal control. Table 1 presents the primers used. The relative gene expression (fold change) was measured with the comparative CT method using U6 as the housekeeping gene and the 2^−∆∆Ct^ formula.

### 2.7. Western Blotting Analysis

The TM3 were cultured in 6 well plates and treated with 0.01 μmol/L. Cells were lysed with RIPA lysis buffer. The cell lysis solution was collected to detect the whole proteins concentration. According to the standard protein curve, each sample was adjusted to the same protein concentration. The extracted proteins were added to sodium dodecyl sulfate polyacrylamide gel and the proteins were separated on SDS–PAGE and then transferred from gel to PVDF membranes. The PVDF membranes were blocked in the 5% milk buffer for 2 h, and then covered with antibody to be kept for overnight in ice box. After the membrane was washed by TBST for 5 times, the secondary antibody was added and the incubation continued for further 2 h. The ECL detection system was used to detect the signal of secondary antibody, and the relative photographic density was analyzed using a gel documentation and analysis software.

### 2.8. Statistical Analysis

Statistical data were analyzed by using a non-parametric, SPSS (Armonk, New York, NY, USA) one-way analysis of variance, with *p  *<  0.05 considered statistically significant. The results are presented as the mean ± standard deviation (SD).

## 3. Results

### 3.1. ZEA Can Stimulate the TM3 Cells Proliferation

**Figure 1:**
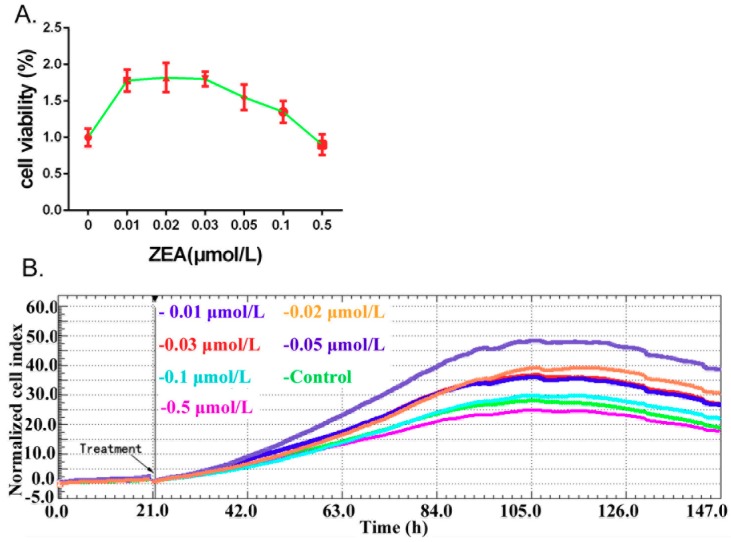
The effects of ZEA on the cell proliferation in TM3 cells. (**A**) Cell viability was detected by using the CCK-8 assay kit. (**B**) Cell proliferation was monitored by using the xCELLigence system.

The effects of ZEA (0, 0.01, 0.02, 0.03, 0.05, 0.1, 0.5 μmol/L) on the cell viability were assessed by using the CCK-8, and cell proliferation was monitored by the xCELLigence system in real time in TM3 cells. The results from CCK-8 (Figure 1A) indicated that compared with the 0 h group, the viability of the TM3 cells was significantly increased in the groups of 0.01, 0.02, 0.03, 0.05 and 0.1 μmol/L. The results from the xCELLigence system indicated (Figure 1B) that after treatment with ZEA, the cell index value was increased in the groups of 0.01, 0.02, 0.03 and 0.05 μmol/L. These data suggested that ZEA, in low concentrations, promotes the growth of TM3 cells.

### 3.2. The Differentially Expressed microRNAs after Treatment with ZEA

**Figure 2:**
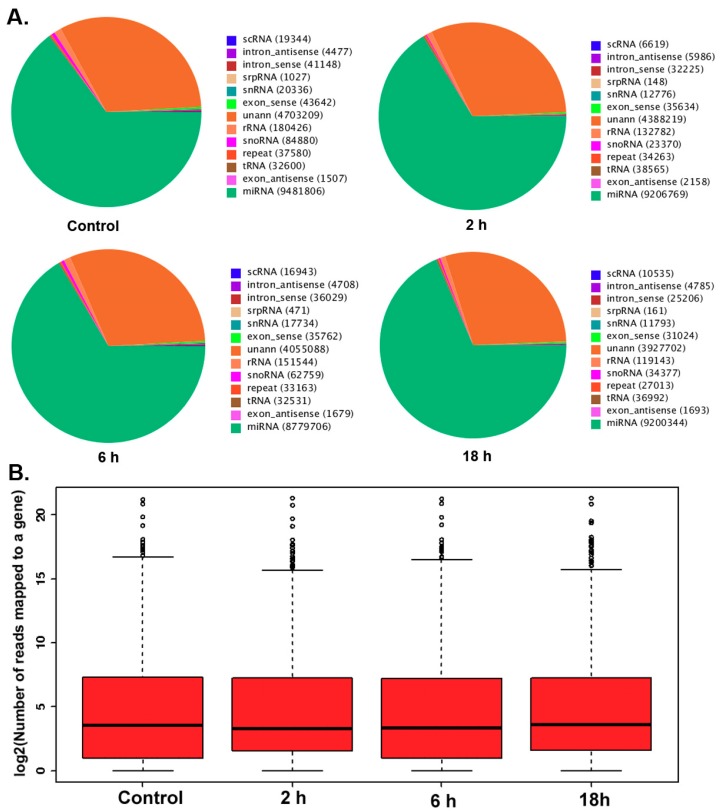
(**A**) The number of small nucleotides in different experimental groups. (**B**) MicroRNA two generation sequencing data box of each experimental group.

MircoRNA deep sequencing analysis was used to identify the differential expression of miRNAs in TM3 cells after treatment with 0.01 μmol/L ZEA for different time periods (0, 2, 6 and 18 h). The pie charts from the Short oLigonucLeotide aLignment program (SOAP) have suggested (Figure 2A) that the total numbers of identified miRNA were 9481806, 920676, 8779706, 9206769 in the groups of 0, 2, 6, 18 h respectively. The data form box-plot showed (Figure 2B) that after normalizing, the log2 ratios of distribution in all samples were similar and symmetric. 

**Figure 3:**
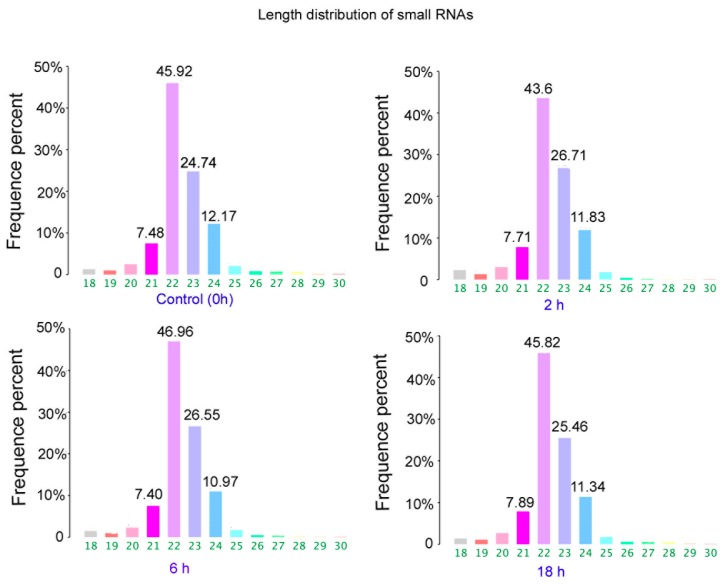
Length distribution of small RNAs in control and ZEA treated groups.

**Figure 4:**
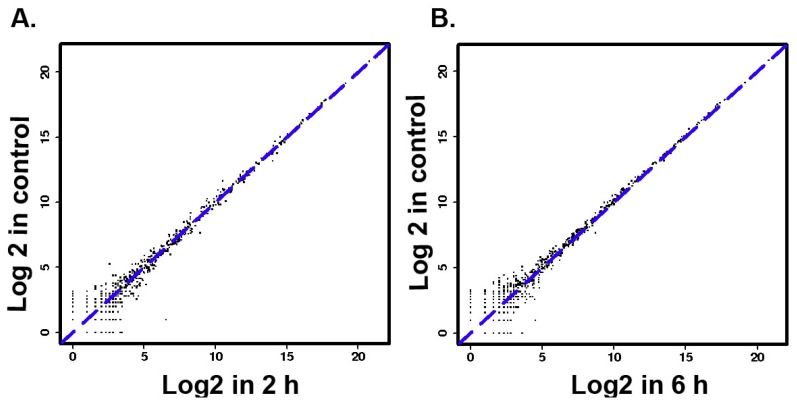

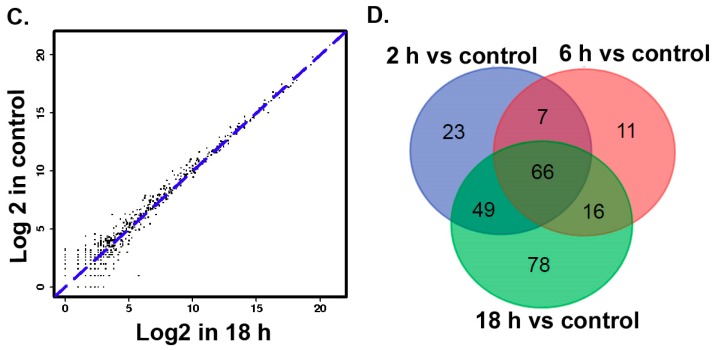
(**A**–**C**) MicroRNAs two generation sequencing data scatter diagram. (**D**) The numbers of significantly different expression miRNAs in different groups.

The results of size distributions have suggested that approximately 90% of these small RNAs were between 21 to 24 nt nucleotides in length (Figure 3). The values of miRNA were compared in logarithmic coordinates to evaluate the consistency of the miRNA expression values of the different groups. The results from scatter plots indicated a good reproduction quality in miRNA expression (Figure 4A). Among these miRNA, 145 miRNAs were significantly (*p* < 0.05) expressed in the 2 h group. Compared with the 0 h group, a total of 100 miRNAs were significantly (*p* < 0.05) expressed in the 6 h group, a total of 209 miRNAs were significantly (*p* < 0.05) expressed in the 18h group. There were 66 identical miRNAs which were significantly expressed in all groups (2 h, 6 h and 18 h) compared with the 0 h group (Figure 4A). The detailed information of these 66 differentially expressed miRNAs is shown in Table 2.

### 3.3. Predicted the Cell Functions and Pathways of miRNAs Target Genes

**Figure 5:**
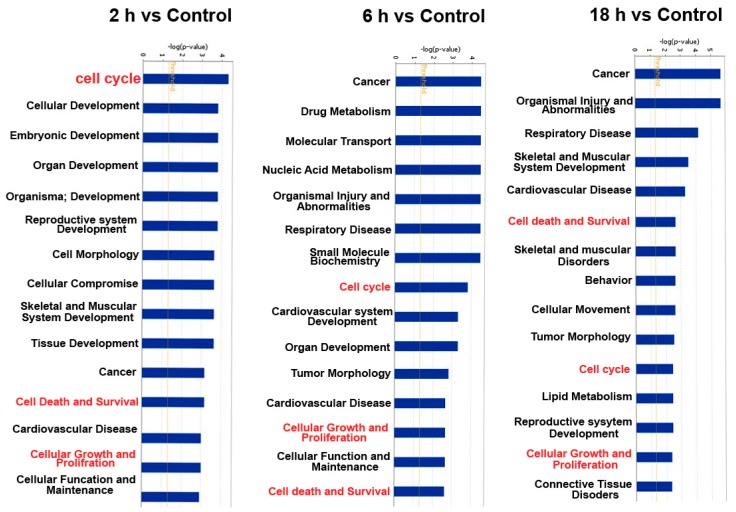
The cellular functions regulated by these 66 differentially expressed miRNAs (*p* <  0.05) as analyzed by IPA.

In order to investigate cellular functions regulated by these differentially expressed genes, the 66 differentially expressed miRNAs were analyzed by using IPA database. The results from IPA showed that these 66 miRNAs are associated with several cellular functions. The top 15 terms of different groups are shown in the Figure 5. The cell functions of target genes in all of groups included cell cycle, cellular growth and proliferation, and cell death and survival. These cell functions have a regulatory effect on cell proliferation.

**Figure 6:**
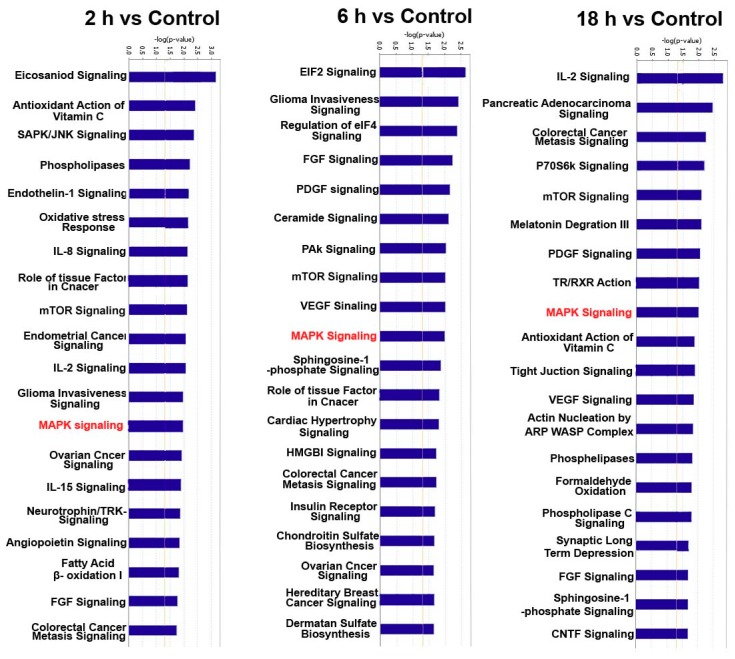
The cell pathways of these 66 differentially expressed miRNAs (*p* <  0.05) as analyzed by IPA.

**Figure 7:**
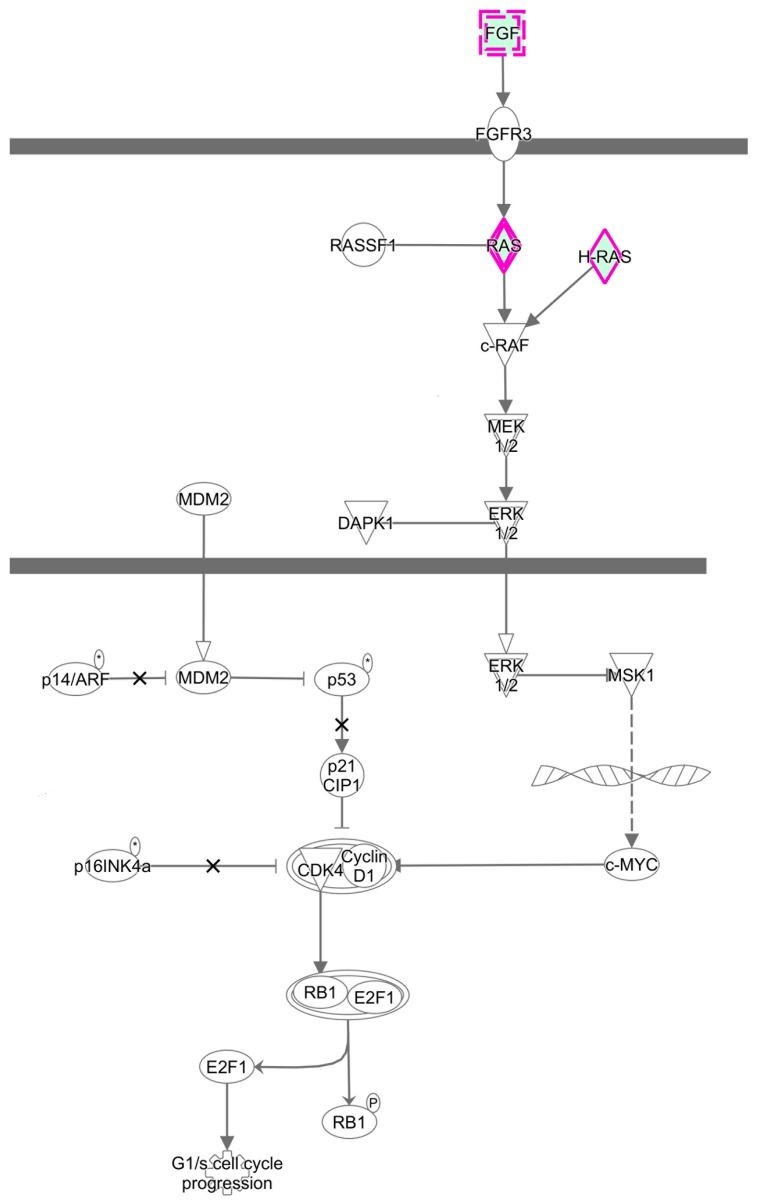
The RAS-RAF-MEK-ERK pathway was predicted to be targeted by differentially expressed miRNA (*p* <  0.05).

To further elucidate the specific functions of these miRNAs target genes, a detailed pathway analysis was performed by using IPA. There were several pathways with high enrichment value. The top 15 terms of each group are shown in the Figure 6. ZEA-affected miRNA target genes have a close relationship with MAPK signaling pathway. These pathways have a regulatory effect on cell proliferation. Additionally, the RAS-RAF-MEK-ERK pathway was predicted to be targeted by these differentially expressed miRNA (Figure 7).

### 3.4. Validation of Differentially Expressed miRNAs by Using QRT-PCR

**Figure 8:**
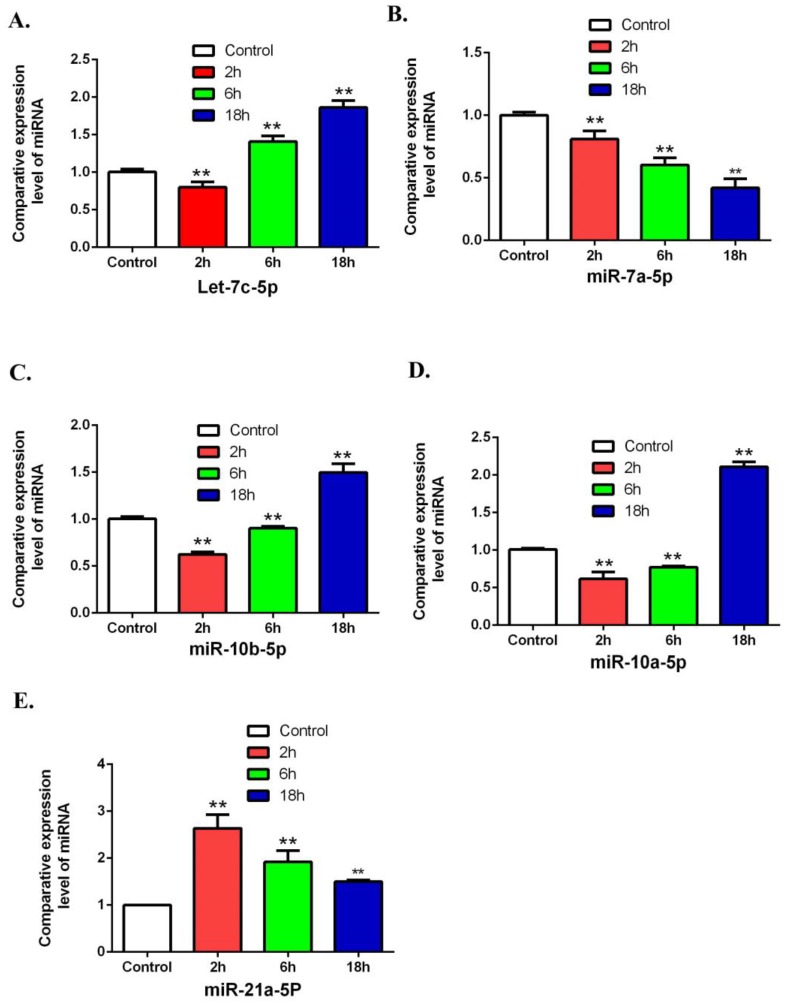
Validating the differentially expression of miRNAs by using QRT-PCR. The expressions of let-7c-5p, miR-7a-5p, miR-10b-5p, miR-10a-5p and miR-21a-5p were detected after treatment with ZEA. (**A**) The effects of ZEA on the expression of let-7c-5p. (**B**) The effects of ZEA on the expression of miR-7a-5p. (**C**) The effects of ZEA on the expression ofmiR-10b-5p. (**D**) The effects of ZEA on the expression of miR-10a-5p. (**E**)The effects of ZEA on the expression of miR-21a-5p. Values represent mean ± SD. Asterisks suggest a statistically significant difference: * *p* < 0.05, ** *p* < 0.01.

To validate the array results in miRNA, this study has randomly selected 5 miRNAs including Let-7c-5p, miR-7a-5p, miR-10b-5p, miR-10a-5p and miR-21a-5p by using QRT-PCR. The results from QRT-PCR showed (Figure 8) that miR-21a-5p was increased in the 2, 6, 18 h groups compared to the control group. MiR-10b-5p and miR-10a-5p were decreased in the 2 and 6 h groups and increased in 18 h groups. let-7c-5p was decreased in the 2 h group and increased in the 6 and 18 h groups. These results are consistent with the micro-array data.

### 3.5. Validating the Effects of ZEA on Cell Cycle

**Figure 9:**
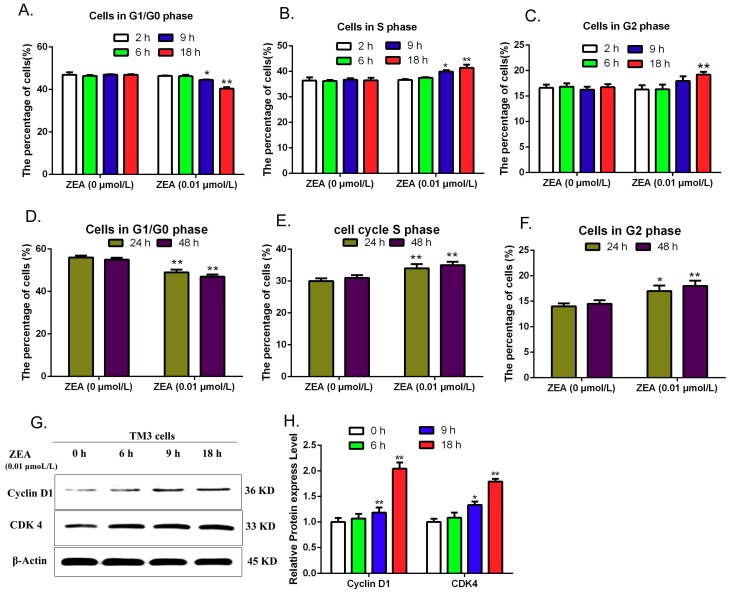
The effects of ZEA on cell cycle in TM3 cells. After treatment with 0.01 μmol/L ZEA for different time (0, 2, 6, 9 and 18 h), cells were harvested to detect the cell cycle distribution and to analyze the expressions of the CyclinD1and CDK4 by western blot. (**A**,**D**) The effects of ZEA on the cell numbers in G1/G0 phase. (**B**,**E**) The effects of ZEA on the cell numbers in S phase. (**C**,**F**) The effect of ZEA on the cell numbers in G2 phase. (**G**,**H**) The effects of ZEA on the expressions of the CyclinD1, and CDK4. Values represent mean ± S.D. Asterisks suggest a statistically significant difference: * *p* < 0.05, ** *p* < 0.01.

IPA software analysis predicated that these ZEA-affected miRNA target genes have a close relationship with cell cycle. Thus, the current study evaluated the effects of ZEA on the cell cycle distribution by using flow cytometric analysis, and the expressions of cell cycle regulatory proteins including Cyclin D1 and Cdk4 were detected by using western blot. The results show (Figure 9A–F) that after treatment with 0.01 μmol/L ZEA for different time (0, 2, 6, 9, 18, 24 and 48 h), the numbers of cells in G1/G0 phases were gradually decreasing and the numbers of cells in S and G2 phases were increased with the delaying of exposing time. Furthermore, the expression of Cyclin D1 and Cdk4 proteins were analyzed by using the western blot (Figure 9G,H). Blots for Cyclin D1 and Cdk4 were semi-quantified by using the Image Lab software. Lanes from left to right represent protein from cells exposed to 0.01 μmol/L ZEA for 0, 6, 9 and 18 h, respectively. The intensities of the Cyclin D1 and Cdk4 bands were normalized with respect to the intensities of the β-actin bands. The expression of Cyclin D1 and Cdk4 proteins increased significantly in the 9 and 18 h groups compared to the control group (0 h). These data revealed that low concentrations of ZEA could promote the progression of cell cycle and affect the expressions of cell cycle regulatory proteins in TM3.

### 3.6. Validating the Effects of the MAPK Signaling Pathway by Uusing Western Blot

**Figure 10:**
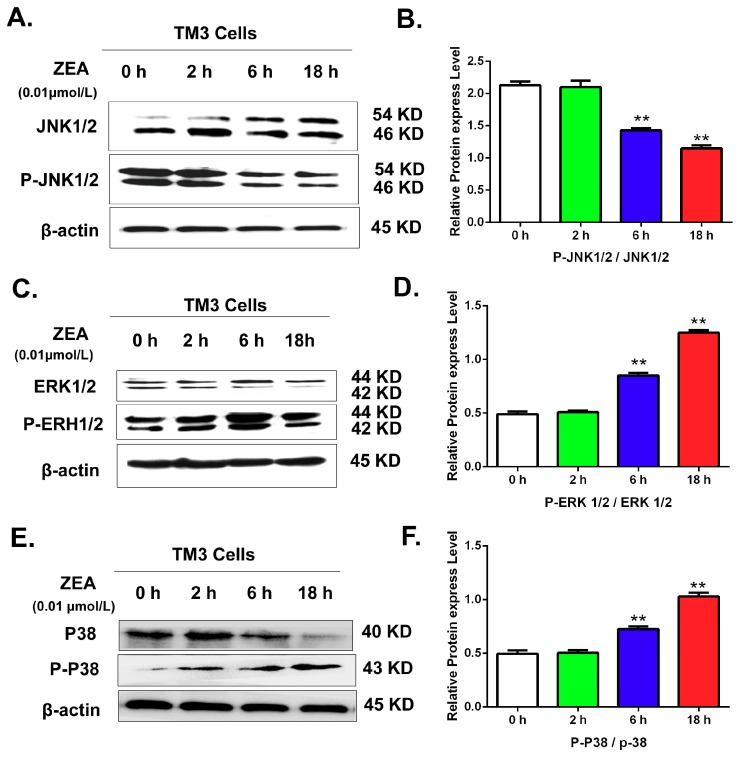
The effects of ZEA on the MAPK family proteins including JNK1/2, p-JNK1/2, ERK1/2, P-ERK1/2, p38 and p-p38. After treated with 0.01 μmol/L ZEA, cells were harvested to detect the rations of JNK1/2/p-JNK1/2, ERK1/2/P-ERK1/2 and p38/p-p38. (**A**) The effects of ZEA on the expressions of the JNK1/2 and p-JNK1/2. (**B**) The rations of JNK1/2/p-JNK1/2. (**C**) The effects of ZEA on the expressions of the ERK1/2 and P-ERK1/2. (**D**) The rations ofERK1/2/P-ERK1/2. (**E**) The effects of ZEA on the expressions of the p38 and p-p38. (**F**) The rations of p38/p-p38.Values represent the mean ± SD. * *p* < 0.05, ** *p* < 0.01 compared to the 0 h group.

IPA software analysis showed that the MAPK pathway was associated with the differentially expressed miRNAs. To further validate the effects of ZEA on MAPK pathways, this study detected the expression of MAPK family proteins by western blot analysis. The results (Figure 10) suggested that after comparison with the 0 h group, the ratio of p-JNK /JNK was significantly decreased. However, the ratios of p-ERK1/2/ERK1/2, and p-p-38/P38 were significantly increased in a time-dependent manner.

## 4. Discussion

The adverse effects of ZEA have been widely investigated in humans, farm animals, poultry, birds and companion animals such as pig, sheep, bovine, horse, chicken, birds, dogs and cats [23,24,25,26,27,28]. ZEA and its derivatives affect the synthesis and secretion of steroid sex hormones through disturbing the cell function of Leydig cells in testis [29,30]. Leydig cells play a crucial role in synthesizing testosterone, and contribute about 95% of the circulatory androgen level [31]. The production of testosterone depends not only on the functions of Leydig cells, but also on their numbers [32]. Thus change of the Leydig cell number may affect the production of testosterone. Furthermore, regulation of cell proliferation is critical for normal development of multicellular organisms, and loss of control ultimately leads to cancer [33]. Defects in this balance are thought to contribute to the development of cancer and other pathological conditions [34]. A study has indicated that ZEA can disturb the dynamic balance between proliferation and apoptosis and causes abnormal regulation of oncogenes in Leydig cells, which may easily induce the translation of normal cells into tumor cells [35]. Numerous studies have demonstrated that aberrant expression of miRNAs is closely associated with proliferation, invasion, metastasis and the prognosis of various cancers [16]. Thus, it is expected to detect the effects of ZEA in TM3 cells. It was indicated that ZEA, in high concentrations, could inhibit the growth of Leydig cells and induce apoptosis [29]. However, other studies have suggested that low doses of ZEA could stimulate cells growth including T47D, MCF-7, KK-1 and granulosa cells [1,10,14,36]. But, the detailed mechanism of ZEA-mediated induction of cell proliferation is still unclear. We speculate that miRNAs may participate in the process of ZEA stimulated cell growth.

Many studies have indicated that high concentrations of ZEA could inhibit cell viability, and a lower concentration of ZEA promote cell proliferation [37]. In order to analyze cell proliferation, lower concentrations of ZEA were used in present study. The results from CCK-8 indicated that, compared to the 0 h group, the viability of the TM3 cells was significantly increased in the groups of 0.01, 0.02, 0.03, 0.05 and 0.1 μmol/L. The results from the xCELLigence system indicated that after treatment with ZEA, the cell index value was increased in the groups of 0.01, 0.02, 0.03 and 0.05 μmol/L. Among these groups the largest increase of cell index was 0.01μmol/L group. Thus, we decided to use dose the 0.01 µmol/L to analyze other parameters. One of our previous studies has analyzed the effects of ZEA on TM3 cell proliferation after treatment with lower doses of ZEA for a loner time (72 h) [35]. In this study, we decided to detect the effects of ZEA on TM3 cell proliferation after treatment for 24 h only. So that, the effects of ZEA on TM3 cell proliferation in both long time and short periods are analyzed, which can provide a theoretical basis for future molecular studies on the toxicology of ZEA.

An increasing volume of literature indicates that miRNAs are involved in regulating the cell proliferation [38]. Micro-RNAs target the noncoding area of mRNA molecules and cause silencing or degradation of transcripts to regulate the expression gene [39]. Micro-RNAs could stimulate cell proliferation and serve as potent oncogenes by silencing several inhibitors of cell cycle [40]. This research revealed that 66 miRNAs were differentially expressed in all groups after treatment with ZEA. The mechanism of ZEN toxicity to humans and animals is complicated. ZEA and its metabolites have structural analogy to estrogen, thus they can bind to estrogen receptors (ERs) and exert the estrogen-like effects [41]. Recent studies have shown that the addition of reproductive hormones including estrogen could affect the expression of miRNAs in TM3 Leydig cells [42]. Furthermore, study has clearly shown that oxidative stress can be a regulator of miRNAs, and can affect the expression of Micro-RNAs [43]. It was reported that ZEA induces the overproduction of reactive oxygen species (ROS) and cause oxidative stress by stimulating leakage electron from the respiratory chain and destroying the antioxidant defense systems in mitochondria [5]. Thus ZEA might affect the expressions of miRNAs through the estrogen-like effects or inducing oxidative stress.

The results from IPA have shown that these 66 miRNAs are found to be associated with several cellular functions. The cell functions of the target genes are associated with cell cycle, cellular growth and proliferation, and cell death and survival. These cell functions have a regulatory effect on cell proliferation. In the present study, our data have validated that ZEA can promote the progression of the cell cycle and increase the expressions of cell cycle-regulating proteins including Cdk4 and Cyclin D1. These data suggested that miRNAs might be involved in the course of ZEA-promoted cell proliferation through regulating the cell cycle. Several studies have shown that ZEA exhibited the carcinogenicity by stimulating cell proliferation [1]. A study has shown that ZEA could promote HCT116 cell proliferation and also promote cell migration and colony formation which are the hallmarks of carcinogenic properties [12]. ZEA was involved in the increasing the risk of hormone-dependent tumors and might exert a crucial role in the increasing incidence of cancer in different organs [44,45]. After exposure to ZEA at environmentally relevant doses, the female rat mammary gland was changed, which might increase the incidence of mammary tumors [46]. After feeding with dietary ZEA for 104 weeks, the weights of liver were increased in both male and female rat and the uterine were increased in females [47]. The alteration and increasing in the weights of these organs is probably a precursor of the carcinogenic properties.

The cell pathways analysis of the differentially expressed genes has indicated that the MAPK signaling pathway is associated to these differentially expressed genes. Our data also confirmed that ZEA can influence the expressions of MAPK family proteins. A study has indicated that the MAPK signaling pathway plays important roles in regulating cell proliferation, and apoptosis, among which the activation of the p38 signaling pathway possibly participates in the course of cell proliferation [48]. There are three major classic MAPK families in mammalian cells: ERK family, p38 family, and SAPK/JNK family. The activation of MAPKs is a key component in signal transduction associated with cell proliferation [49]. The p38 MAPK family consists of four different isoforms including α, β, δ, and γ. α and β isoforms are ubiquitously expressed, whereas γ expression is found predominantly in skeletal muscle, and δ expression is enriched in the lung, kidney, testis, pancreas, and small intestine [50]. Activation of p38 is implicated in inflammation, cell growth control, cell differentiation, cell migration, and apoptosis [51]. Evidence has accumulated showing that p38 can have non-classical roles in proliferation and survival programs [52]. The serine/threonine MAPK p38 is activated through phosphorylation at the Thr180-Gly-Tyr182 motif by MKK3, MKK4 and MKK6. Phosphorylated p38 activates a wide range of substrates, leading to diverse responses, such as cell differentiation, cell cycle, apoptosis, senescence, cytokine production, regulation of RNA splicing and inflammatory responses [53,54]. In this study, after treatment with ZEA, the expression of nonphosphorylated p-38 was decreased, and the expression of phosphorylated p-38 was increased. Such data suggested that ZEA promotes the activation of p-38 and stimulates the phosphorylation of P-38. A study showed that ERK1/2 regulates cell growth and differentiation through the ERK/MAPK signaling pathway as a protein kinase [55]. These data suggested that miRNAs might participate in the course of ZEA promoted cell proliferation through MAPK pathways.

It was indicated that RAS-RAF-MEK-ERK pathway could participate in the regulation of cell cycle progression and apoptosis in many cell types, and be involved in maintaining cell survival by activating molecules of apoptosis [56]. RAS-RAF-MEK-ERK regulates the cellular growth and survival through integrating extracellular signals and coordinating a suitable response. The RAF genes can prevent the apoptosis and affect cell cycle progression in hematopoietic cells [57]. Our data have indicated that the RAS-RAF-MEK-ERK pathway is associated with these differentially expressed miRNA genes suggesting that miRNAs might participate in the course of ZEA promoted cell proliferation through RAS-RAF-MEK-ERK pathway.

## 5. Conclusions

Differentially expressed miRNAs were identified after treatment with ZEA in TM3 Leydig cells. 66 miRNAs and target genes were identified to exhibit differential expression. Analyzing the cell functions and pathways of miRNAs target genes showed that miRNA and target genes are closely related to the cell proliferation induced by ZEA. The miRNAs might be involved in the course of ZEA promoted cell proliferation through regulating cell cycle. The miRNAs might participate in ZEA promoted cell proliferation through MAPK and RAS-RAF-MEK-ERK pathways. This study provides a molecular basis and new insights into the toxicological mechanisms of ZEA.

## Figures and Tables

**Table 1:** The information of primers used in this RT-qPCR.

Name	Primer	Primer Sequence
miR-21a-5p	RT primer	UAGCUUAUCAGACUGAUGUUGA
	Forward primer	GCAGTAGCTTATCAGACTGATA
	Reverse primer	GGTCCAGTTTTTTTTTTTTTTTCAAC
miR-10a-5p	RT primer	UACCCUGUAGAUCCGAAUUUGUG
	Forward primer	GCAGTACCCTGTAGATCCGA
	Reverse primer	GGTCCAGTTTTTTTTTTTTTTTCAC
miR-10b-5p	RT primer	UACCCUGUAGAACCGAAUUUGUG
	Forward primer	CAGTACCCTGTAGAACCGA
	Reverse primer	GGTCCAGTTTTTTTTTTTTTTTCAC
miR-7a-5p	RT primer	UGGAAGACUAGUGAUUUUGUUGU
	Forward primer	CGCAGTGGAAGACTAGTGA
	Reverse primer	GTCCAGTTTTTTTTTTTTTTTACAACA
let-7c-5p	RT primer	UGAGGUAGUAGGUUGUAUGGUU
	Forward primer	GCAGTGAGGTAGTAGGTTGT
	Reverse primer	GGTCCAGTTTTTTTTTTTTTTTAACCA
U6	Forward primer	GGAACGATACAGAGAAGATTAGC
	Reverse primer	TGGAACGCTTCACGAATTTGCG

**Table 2:** Differentially expressed (*p* < 0.05) miRNA after treatment with 0.01 μmol/L ZEA for different time (0, 2, 6 and 18 h).

miRNA Title	2 h VS. Control		6 h VS. Control		18 h VS. Control	
	Fold Changes log2	*p*-Value	Fold Changes log2	*p*-Value	Fold Changes log2	*p*-Value
mmu-let-7a-5p	−0.12	1.04 × 10^−184^	−0.04	2.06 × 10^−21^	0.14	1.94 × 10^−251^
mmu-let-7b-3p	0.45	0.00	0.42	9.89 × 10^−4^	−0.91	1.07 × 10^−8^
mmu-let-7b-5p	−0.10	0.00	−0.03	2.13 × 10^−58^	−0.39	0.00
mmu-let-7c-5p	−0.01	0.00	0.06	9.80 × 10^−134^	0.14	0.00
mmu-let-7e-5p	−0.07	1.01 ×10^−29^	−0.03	6.96 × 10^−7^	−0.14	1.55 × 10^−109^
mmu-let-7f-5p	−0.17	0.00	−0.12	5.31 × 10^−152^	0.28	0.00
mmu-let-7i-3p	5.56	9.77 × 10^−24^	3.63	3.48 × 10^−6^	4.80	3.40 × 10^−14^
mmu-let-7i-5p	−0.05	0.00	0.02	1.73 × 10^−57^	−0.07	0.00
mmu-miR-100-5p	−0.13	1.19 × 10^−132^	−0.14	1.82 × 10^−166^	0.40	0.00
mmu-miR-10a-5p	−0.17	0.00	−0.02	5.98 × 10^−9^	0.38	0.00
mmu-miR-10b-5p	−0.21	6.45 × 10^−254^	−0.07	1.36 × 10^−26^	0.31	0.00
mmu-miR-1198-5p	−0.19	0.00	−0.08	2.27 × 10^−4^	−0.27	1.12 × 10^−30^
mmu-miR-125a-5p	−0.03	3.88 × 10^−4^	−0.07	6.84 × 10^−19^	0.03	2.28 × 10^−5^
mmu-miR-125b-5p	0.06	0.00	−0.05	5.03 × 10^−5^	0.19	9.56 × 10^−73^
mmu-miR-128-3p	0.06	2.44 × 10^−3^	0.13	3.37 × 10^−10^	−0.25	7.47 × 10^−31^
mmu-miR-139-3p	3.36	0.00	3.70	7.45 × 10^−4^	3.04	1.21 × 10^−2^
mmu-miR-140-3p	0.30	5.09 × 10^−24^	0.13	3.49 × 10^−5^	0.36	9.26 × 10^−36^
mmu-miR-143-3p	0.10	0.00	0.07	7.51 × 10^−12^	0.36	5.60 × 10^−299^
mmu-miR-148a-3p	−0.06	2.76 × 10^−33^	0.15	8.57 × 10^−191^	0.18	4.63 × 10^−275^
mmu-miR-149-3p	1.28	0.00	1.14	1.15 × 10^−21^	−1.40	2.31 × 10^−14^
mmu-miR-151-3p	−0.07	3.47 × 10^−17^	0.08	1.75 × 10^−19^	−0.17	1.94 × 10^−85^
mmu-miR-16-1-3p	−0.20	0.00	−0.52	1.05 × 10^−18^	−0.94	1.75 × 10^−50^
mmu-miR-182-3p	−0.59	6.70 × 10^−5^	−0.39	6.57 × 10^−3^	−1.57	3.28 × 10^−19^
mmu-miR-182-5p	−0.11	0.00	−0.06	7.50 × 10^−14^	0.12	6.94 × 10^−67^
mmu-miR-183-5p	−0.05	5.08 × 10^−20^	0.02	2.03 × 10^−4^	0.10	4.05 × 10^−74^
mmu-miR-196b-5p	−0.15	0.00	−0.07	5.29 × 10^−4^	0.09	1.02 × 10^−5^
mmu-miR-199a-3p	0.29	1.22 × 10^−69^	0.07	3.34 × 10^−5^	0.31	5.12 × 10^−80^
mmu-miR-199a-5p	−0.48	0.00	−0.22	4.46 × 10^−75^	0.93	0.00
mmu-miR-199b-3p	0.29	1.92 × 10^−69^	0.07	5.92 × 10^−5^	0.31	1.40 × 10^−79^
mmu-miR-206-3p	−0.22	0.00	−0.20	6.43 × 10^−9^	−0.83	8.43 × 10^−110^
mmu-miR-21a-5p	0.15	0.00	0.04	5.92 × 10^−210^	0.03	2.08 × 10^−114^
mmu-miR-221-5p	−0.75	0.00	−0.32	5.85 × 10^−11^	−1.10	2.09 × 10^−85^
mmu-miR-222-3p	−0.02	4.47 × 10^−4^	−0.10	5.31 × 10^−63^	−0.46	0.00
mmu-miR-23a-3p	0.55	0.00	0.26	5.69 × 10^−5^	0.38	1.56 × 10^−9^
mmu-miR-24-3p	0.17	4.78 × 10^−33^	0.08	3.27 × 10^−7^	−0.05	4.25 × 10^−4^
mmu-miR-25-5p	−0.12	0.01	−0.38	6.66 × 10^−16^	−0.69	1.64 × 10^−44^
mmu-miR-27a-3p	0.83	0.00	0.40	4.16 × 10^−88^	0.28	6.97 × 10^−44^
mmu-miR-27a-5p	0.13	0.00	−0.13	5.94 × 10^−8^	−0.80	2.57 × 10^−196^
mmu-miR-27b-3p	0.57	5.61 × 10^−287^	0.27	7.54 × 10^−59^	0.31	2.35 × 10^−80^
mmu-miR-28a-3p	−0.13	0.00	−0.13	1.31 × 10^−5^	0.14	7.11 × 10^−7^
mmu-miR-296-3p	0.21	8.10 × 10^−38^	−0.05	2.18 × 10^−3^	−0.34	1.19 × 10^−82^
mmu-miR-30c-2-3p	−0.35	0.00	−0.35	2.65 × 10^−13^	−0.60	6.46 × 10^−33^
mmu-miR-30d-5p	−0.09	4.16 × 10^−36^	−0.12	3.95 × 10^−67^	0.10	6.42 × 10^−51^
mmu-miR-30e-3p	−0.16	0.00	−0.13	1.28 × 10^−5^	−0.18	4.95 × 10^−10^
mmu-miR-342-5p	−1.12	1.39 × 10^−143^	−0.32	7.78 × 10^−17^	−1.09	4.07 × 10^−138^
mmu-miR-351-3p	−0.41	0.00	−0.44	2.25 × 10^−8^	−0.77	2.97 × 10^−20^
mmu-miR-365-1-5p	−0.57	7.98 × 10^−13^	−0.40	3.68 × 10^−7^	−1.04	2.44 × 10^−33^
mmu-miR-365-2-5p	−0.51	0.00	−0.24	2.82 × 10^−7^	−1.13	3.12 × 10^−95^
mmu-miR-374b-5p	0.36	5.55 × 10^−9^	0.20	1.95 × 10^−3^	0.27	1.76 × 10^−5^
mmu-miR-378a-3p	0.17	0.00	−0.07	1.08 × 10^−6^	0.11	2.11 × 10^−17^
mmu-miR-423-5p	−0.10	3.14 × 10^−38^	−0.16	5.19 × 10^−92^	−0.44	0.00
mmu-miR-486a-3p	−0.29	0.00	−0.18	3.14 × 10^−4^	−0.55	6.25 × 10^−26^
mmu-miR-501-3p	−0.43	3.95 × 10^−45^	−0.20	2.82 × 10^−11^	0.46	4.11 × 10^−71^
mmu-miR-503-5p	0.45	0.00	0.21	1.04 × 10^−8^	0.27	3.56 × 10^−14^
mmu-miR-532-5p	−0.16	7.28 × 10^−52^	−0.05	3.51 × 10^−7^	0.39	0.00
mmu-miR-574-5p	−0.47	0.00	−0.07	3.05 × 10^−7^	−0.59	0.00
mmu-miR-615-3p	0.26	9.94 × 10^−97^	0.16	2.72 × 10^−34^	0.11	2.35 × 10^−17^
mmu-miR-615-5p	−0.73	0.00	−0.30	3.96 × 10^−6^	−0.75	1.29 × 10^−26^
mmu-miR-669c-5p	−0.28	9.79 × 10^−78^	−0.05	1.32 × 10^−3^	−0.22	4.30 × 10^−50^
mmu-miR-671-3p	−0.42	0.00	−0.23	1.00 × 10^−5^	−1.00	1.11 × 10^−66^
mmu-miR-7a-5p	−0.03	1.80 × 10^−8^	−0.19	0.00	−0.47	0.00
mmu-miR-877-5p	−0.44	0.00	−0.34	8.23 × 10^−7^	−1.55	5.50 × 10^−76^
mmu-miR-92b-5p	−0.92	2.48 × 10^−20^	−0.40	1.58 × 10^−5^	−1.19	4.37 × 10^−30^
mmu-miR-93-5p	−0.12	0.00	−0.25	4.43 × 10^−9^	0.09	1.52 × 10^−2^
mmu-miR-98-5p	−0.08	3.33 × 10^−6^	−0.07	4.45 × 10^−5^	−0.16	5.41 × 10^−19^
mmu-miR-99b-3p	−0.10	0.00	−0.11	1.33 × 10^−9^	−0.70	1.32 × 10^−274^

## Abbreviations

**Abbreviation**	**Full name**
3α-HSD	3α-Hydroxysteroid dehydrogenase
3β -HSD	3β–Hydroxysteroid dehydrogenase
α-ZEA	α-Zearalenol
α-ZAL	α-Zearalanol
β-ZAL	β-Zearalanol
β-ZEA	β-Zearalenol
CCK8	Cell counting kit-8 assay
Cdk4	Cyclin-dependent kinase 4
Cyclin D1	Cyclin-delta-1
ERK	Extracellular signal-regulated kinase
JNK	c-Jun N-terminal kinases
MAPK	Mitogen-activated protein kinase
MEK	Dual specificity mitogen-activated protein kinase
miRNA	MicroRNA
RAF	Serine/threonine-protein kinase
RTCA	Real-time cell analysis
SOAP	Short oLigonucLeotide aLignment program
ZAN	Zearalanone
ZEA	Zearalenone
